# Serum exosomal microRNA-144-3p: a promising biomarker for monitoring Crohn's disease

**DOI:** 10.1093/gastro/goab056

**Published:** 2021-12-23

**Authors:** Peng Chen, Shanshan Huang, Qiao Yu, Kang Chao, Ying Wang, Gaoshi Zhou, Xiaojun Zhuang, Zhirong Zeng, Minhu Chen, Shenghong Zhang

**Affiliations:** 1 Department of Gastroenterology, The First Affiliated Hospital, Sun Yat-sen University, Guangzhou, P. R. China; 2 Department of Gastroenterology, The Second Affiliated Hospital of Zhejiang University School of Medicine, Hangzhou, P. R. China; 3 Department of Gastroenterology, The Sixth Affiliated Hospital, Sun Yat-sen University, Guangzhou, P. R. China

**Keywords:** Crohn's disease, microRNA-144-3p, serum biomarker, exosome

## Abstract

**Background:**

Crohn’s disease (CD) has a tendency for recurrence and requires adequate monitoring and personalized treatment. Since endoscopy is considerably invasive, serum biomarkers are required as alternatives for CD monitoring. Toward this, exosomal microRNAs (miRNAs) may serve as promising candidates. In this study, we aimed to assess the role of serum exosomal microRNA-144-3p (miR-144-3p) as a biomarker for CD monitoring.

**Methods:**

We prospectively recruited 154 patients without a history of surgery (Cohort 1) and 75 patients who were to undergo intestinal resection (Cohort 2). Serum samples were collected from Cohort 1 before colonoscopy and from Cohort 2 before surgery and during post-operative colonoscopic examination. The serum levels of exosomal miR-144-3p were measured using quantitative reverse-transcription polymerase chain reaction (PCR). Correlations between relative exosomal miR-144-3p levels, disease activity, and disease behavior were analysed. The area under the receiver-operating characteristic curve (AUC) was used to assess the predictive value of exosomal miR-144-3p regarding mucosal activity and post-operative recurrence.

**Results:**

A 3.33-fold increase in serum exosomal miR-144-3p levels was recorded in patients with CD compared with those in healthy controls (*P* < 0.001). The exosomal miR-144-3p levels were positively correlated with the simple endoscopic score of CD (ρ = 0.547, *P* < 0.001) as well as the Rutgeerts score (ρ = 0.478, *P* < 0.001). Elevated exosomal miR-144-3p levels were correlated with the penetrating disease with high specificity (100% [95% confidence interval, 95.1%–100%]). The accuracy of exosomal miR-144-3p for identifying post-operative recurrence was higher than that of C-reactive protein (CRP) (AUC, 0.775 vs 0.639; *P* < 0.001).

**Conclusions:**

Serum exosomal miR-144-3p is a reliable biomarker of mucosal inflammation and penetrating CD. It may identify endoscopic CD recurrence after intestinal resection with higher accuracy than CRP testing.

## Introduction

Crohn’s disease (CD), a subtype of inflammatory bowel disease (IBD), is a chronic inflammatory disease of the gastrointestinal tract, which arises from complex interactions among genetic susceptibility, environmental factors, microbial dysbiosis, and immune responses [1]. Effective control of inflammation is critical to prevent disease progression and long-term complications [2]. Moreover, early identification of disease flare-up and complications in patients with mild symptoms is essential to achieve better treatment responses [3]. The treat-to-target concept (based on mucosal healing) is commonly accepted. Adequate disease monitoring is the key to making timely treatment adjustments [4]. Moreover, nearly 70% of patients with CD require intestinal surgery at least once in their lifetime [5]. Therefore, it is important to predict post-operative recurrence and make timely decisions. Ileocolonoscopy is a standard criterion for evaluating and monitoring mucosal inflammation; however, this method is costly and associated with risks and discomfort, especially in post-surgical-resection patients [6, 7]. C-reactive protein (CRP), the erythrocyte sedimentation rate (ESR), and fecal calprotectin are the most common minimally or non-invasive biomarkers of inflammation used in the clinical diagnosis of CD. However, these markers cannot accurately predict mucosal active disease due to their low negative predictive value (NPV) [8]. CRP production varies between patients owing to genetic heterogeneity, and normal CRP levels can be observed under inflammatory conditions [3]. Fecal-calprotectin levels observed may be biased due to differences regarding detection platforms, collection techniques, stool characteristics, and other factors, thus lacking technical and clinical reliability for monitoring the endoscopic activity of CD [9]. Therefore, reliable non-invasive methods to monitor CD are urgently required.

Exosomes are nanovesicles with a diameter of 40–160 nm, which are generated in the endosomal system by different cell types. Importantly, exosomes mediate gene expression and cellular function by conveying signals and molecules to distal cells. Exosomes carry specific functional proteins, metabolites, and nucleic acids, including DNA and various types of RNAs [10]; prior research has shown that ∼80% of exosomal RNAs (exoRNAs) are microRNAs (miRNAs) [11]. miRNAs are short (∼18–24 nucleotides) non-coding RNAs, which regulate gene expression through mRNA degradation and the inhibition of translation. Dysregulation of certain miRNAs has been causally associated with several autoimmune and neoplastic diseases [12, 13]. Exosomal miRNAs may promote inflammatory responses by modulating the functions of immune cells and the NF-κB signaling pathway in patients with IBD [14, 15]. Furthermore, several studies have proposed the clinical significance of miRNAs as non-invasive biomarkers for various types of cancers, inflammatory diseases, and age-related diseases [16–20]. To date, the use of circulating miRNAs as IBD biomarkers is mostly limited to circulating total cell-free miRNAs [21–25]. Circulating cell-free miRNAs include exosomal miRNAs actively secreted by cells, as well as miRNAs bound to protein complexes that are passively released by apoptotic and necrotic cells. Therefore, exosomal miRNAs may be accurate markers of pathological changes and may have higher disease specificity than cell-free miRNAs [26, 27]. Moreover, exosomal miRNAs are remarkably stable against degradation during long-term storage, as they are protected by the lipoprotein complex structure of the exosome. Conversely, circulating miRNAs not encapsulated in vesicles can easily be degraded by proteinase K and ribonucleases (RNases), limiting the accuracy and reproducibility of serum miRNAs as biomarkers [28].

The predictive value of serum exosomal miRNAs in patients with CD has not been studied to date. Therefore, we assessed whether serum exosomal miRNAs could be used as non-invasive biomarkers for IBD monitoring. miR-144-3p was selected from our exosomal miRNA microarray data of serum samples and its predictive value for endoscopic activity was explored in a prospective study with two independent cohorts. Correlations between exosomal miR-144-3p levels and the CD-activity index (CDAI), the simple endoscopic score for CD (SES-CD), and the Rutgeerts score were evaluated. Our findings suggest that the expression of circulating exosomal miR-144-3p may be a reliable biomarker to detect endoscopically active disease and early post-operative recurrence in patients with CD.

## Patients and methods

### Study subjects

Between May 2017 and May 2019, 229 patients with CD, prospectively admitted in the First Affiliated Hospital of Sun Yat-sen University (Guangzhou, China), the Second Affiliated Hospital of Zhejiang University School of Medicine (Hangzhou, China), and the Sixth Affiliated Hospital of Sun Yat-sen University (Guangzhou, China), were recruited. One hundred age- and sex-matched healthy individuals were also recruited and underwent colonoscopy for routine health check-ups (including those who had a family history of polyposis and colorectal cancer); none of them had any digestive symptoms and endoscopically abnormal manifestations. Patients with CD were diagnosed based on clinical presentation, ileocolonoscopy, histology, radiology, and laboratory tests, with complete clinical data and regular follow-up examinations [29]. Patients with concurrent autoimmune diseases, recent infections, malignant diseases, and pregnancy were excluded. In addition, demographic data, disease duration, and previous treatments of all patients were recorded. This study was approved by the research ethics committees of the First Affiliated Hospital of Sun Yat-sen University ([2018]52 and [2021]356), the Second Affiliated Hospital of Zhejiang University School of Medicine (2018–132), and the Sixth Affiliated Hospital of Sun Yat-sen University (2016ZSLYEC-053). Written informed consent was obtained from all participants.

### Study design

We collected serum samples from three patients with active CD and three healthy volunteers, and performed exosome miRNA microarray analysis (Supplementary Figure 1). Significantly differentially expressed miRNAs (fold change > 2; *P* < 0.001) were selected and compared with previous miRNA microarray data of the colon tissue [24]; miR-144-3p was highly upregulated in the serum and colon tissue of patients with CD, which was the single congruent result of the two miRNA microarrays. Previous studies have also reported overexpression of miR-144 in the colonic mucosa of CD patients [30]; therefore, we assessed the value of serum exosomal miR-144-3p for CD monitoring. Two independent cohorts were enrolled: Cohort 1 included 154 CD patients without a history of gastrointestinal surgery and 100 healthy controls; Cohort 2 included 75 CD patients who underwent intestinal resection. Patients in Cohort 1 underwent ileocolonoscopy at their first visit, when symptoms flared up, or during regular reexamination. Patients in Cohort 2 were followed up for 18 months; they underwent ileocolonoscopy after surgery when suffering from the digestive symptoms of disease recurrence or periodic reexamination within 18 months. The end point was post-operative endoscopic recurrence. In Cohort 1, the correlation between the serum exosomal miR-144-3p levels and the disease activity (CDAI and SES-CD) was explored, and the correlation between the latter and the serum total cell-free miR-144-3p was evaluated; correlations of serum exosomal miR-144-3p and medication, disease behavior, and disease location were also evaluated. In Cohort 2, correlations between serum exosomal miR-144-3p levels and endoscopic recurrence (Rutgeerts score ≥ i2) were explored. The predictive value of miR-144-3p levels for endoscopic recurrence was compared with that of CRP and ESR.

### Disease-activity assessment and blood-sample collection

Endoscopic activity was evaluated according to SES-CD or Rutgeerts scores for CD by experienced physicians who were blinded to clinical information and test results. An SES-CD score of ≥3 was considered to indicate endoscopically active CD [31]. Post-operative endoscopic recurrence refers to the reappearance of mucosal lesions after surgery, defined by a Rutgeerts score of ≥i2 (moderate to severe lesions) [32]. Blood samples were collected for exoRNA extraction and laboratory tests, including those for the inflammatory markers CRP and ESR. In Cohort 1, blood samples were collected within 1 week prior to ileocolonoscopy. In Cohort 2, blood samples were collected from patients who underwent intestinal resection before surgery within 1 week of the procedure; additionally, blood samples were also collected within 1 week prior to post-operative colonoscopy. Laboratory parameters CRP and ESR were recorded. Evaluation of clinical disease activity and collection of blood samples were performed simultaneously. Clinical disease activity was evaluated prospectively using the CDAI. A CDAI score of ≥150 points was considered as clinically active disease [29]. We categorized disease location and phenotype using the Montreal classification [33].

### Exosomal RNA extraction and quantitative real-time polymerase chain reaction (RT-qPCR)

Blood samples were drawn in blood-collection tubes with a clot activator and were processed within 1 h. Samples were centrifuged at 1,900 × *g* and 4°C for 10 mins, and further passed through a 0.8-µm filter to remove cellular material and retain exosomes. Serum samples were frozen in aliquots at −80°C prior to RNA extraction. ExoRNA was extracted using an exoRNeasy Serum/Plasma Maxi Kit (Qiagen, Hilden, Germany) following the manufacturer’s instructions, showing high purity and recovery yield of exoRNA without isolation of serum exosomes [34, 35]. Serum total cell-free miRNAs were isolated using a miRNeasy Serum/Plasma Kit (Qiagen). Mature miRNAs were reverse-transcribed to cDNA following polyadenylation (miScript II RT Kit, Qiagen). RT-qPCR was carried out on a CFX96 Touch Real-Time PCR Detection System (Bio-Rad, Hercules, CA, USA) using a miScript SYBR Green PCR Kit (Qiagen). U6sn was used as an internal control to normalize the miRNA expression [26].

### Statistical analyses

IBM SPSS Statistics 20 (IBM, Armonk, NY, USA) was used for data analyses. A Durbin−Watson test was used to analyse data distribution, the 2^−ΔΔCt^ method was used for relative miR-144-3p quantification, and a Kruskal–Wallis test was used for comparisons among multiple groups. In addition, a Mann–Whitney *U* test was used for pairwise comparisons of non-normally distributed variables. Correlations between two variables were evaluated using the Spearman rank correlation coefficient (ρ) for nonparametric correlations. Receiver-operating characteristic (ROC) curves were used to estimate the accuracy of disease-behavior, mucosal-activity, and endoscopic-recurrence assessments. A chi-square test was applied to compare sensitivity and specificity, and the DeLong’s method was used to compare the areas under ROC curves (AUC) (MedCalc version 19.6.1, MedCalc Software bv, Ostend, Belgium). The tests were two-tailed and statistical significance is reported at *P* < 0.05. Bonferroni corrections were used to adjust significance levels after multiple testing [36].

## Results

### Elevated serum exosomal miR-144-3p in patients with CD

**Figure 1.**
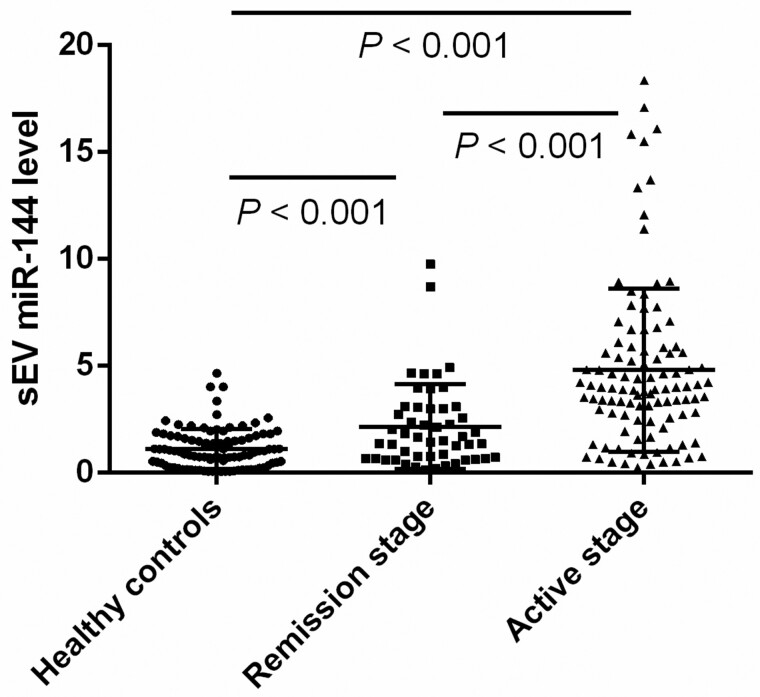
Expression of serum exosomal miR-144-3p in healthy controls, Crohn’s disease (CD) patients in endoscopic remission, and CD patients with endoscopic active disease. The levels of serum exosomal miR-144-3p were considerably higher in patients with active CD than those in healthy individuals or patients with quiescent disease. CD, Crohn’s disease; sEV, small extracellular vesicle.

Baseline demographics of CD patients and healthy controls in Cohort 1 are shown in Table 1; no significant differences in demographic characteristics, including age and sex, were observed between the two groups. A Kruskal–Wallis test showed no statistical difference in the total serum miR-144-3p and exosomal miR-144-3p levels among patients who took different medications (*P* = 0.861 and *P* = 0.710, respectively). Disease duration was not correlated with total serum miR-144-3p and exosomal miR-144-3p levels (*ρ* = −0.122, *P* = 0.132 and *ρ* = 0.018, *P* = 0.829, respectively). Total serum miR-144-3p and exosomal miR-144-3p levels were significantly higher in CD patients than in healthy controls (fold change _[__CD/HC__]_: 2.24 and 3.33, respectively; *P* < 0.001 each). Total serum miR-144-3p levels were not significantly correlated with serum-exosome-derived miR-144-3p levels. Serum exosomal miR-144-3p levels were 3.94- and 2.12-fold higher in patients with active CD than those in healthy controls and patients with endoscopic remission, respectively (*P* < 0.001; Figure 1), while no significant difference was observed in the serum total miR-144-3p levels between mucosal active disease and quiescent disease (*P* = 0.116).

**Table 1.** Demographic and clinical characteristics of 254 participants in Cohort 1

Characteristic	CD patients (*n* = 154)	Healthy controls (*n* = 100)
Male, *n* (%)	99 (64.3)	60 (60.0)
Age, years, median (IQR)	31.5 (23.0–37.2)	28.0 (23.0–30.0)
Disease duration, months, median (IQR)	36.0 (12.0–78.0)	
Clinically active, *n* (%)	79 (51.3)	
Endoscopically active, *n* (%)	104 (67.5)	
Disease phenotype, *n* (%)		
Inflammatory (B1)	71 (46.1)	
Stricturing (B2)	50 (32.5)	
Penetrating (B3)	33 (21.4)	
Perianal disease, *n* (%)	61 (39.6)	
Disease location, *n* (%)		
Terminal ileum (L1)	40 (26.0)	
Colon (L2)	7 (4.5)	
Ileocolon (L3)	107 (69.5)	
Upper gastrointestinal disease (L4)	12 (7.8)	
Medication, *n* (%)		
5-ASA	31 (20.1)	
Corticosteroids	12 (7.8)	
Azathioprine	49 (31.8)	
Mercaptopurine	4 (2.6)	
Methotrexate	9 (5.8)	
Thalidomide	15 (9.7)	
Anti-TNF-α therapy	34 (22.1)	
Vedolizumab	2 (1.3)	

CD, Crohn’s disease; IQR, interquartile range; 5-ASA, 5-aminosalicylic acid; TNF, tumor necrosis factor.

### Predictive value of serum exosomal miR-144-3p for endoscopically active disease

**Figure 2.**
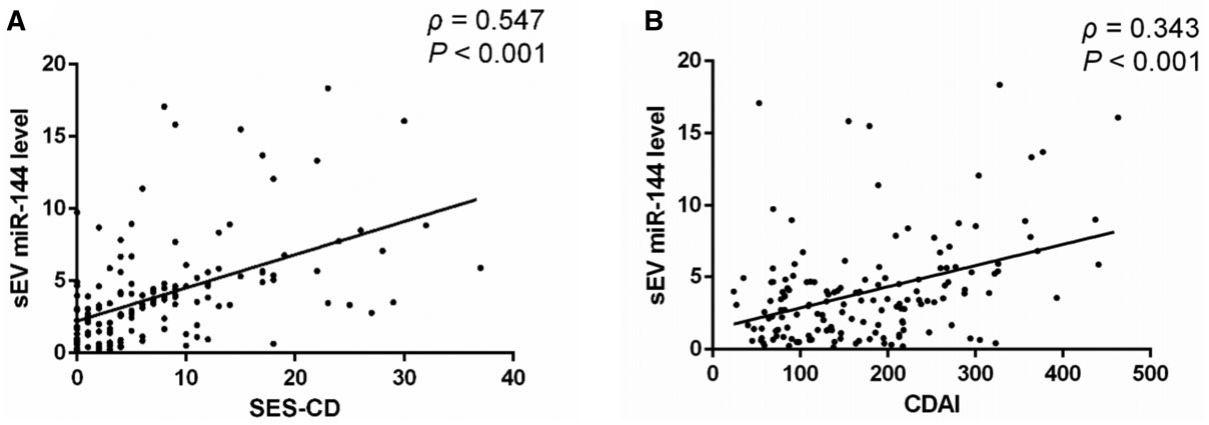
Correlations between serum exosomal miR-144-3p and CDAI, SES-CD in patients with CD. (A) Spearman correlation analysis of miR-144-3p with SES-CD. (B) Spearman correlation analysis of miR-144-3p with CDAI. CD, Crohn’s disease; CDAI, CD-activity index; SES-CD, simple endoscopic score for CD; sEV, small extracellular vesicle.

**Figure 3.**
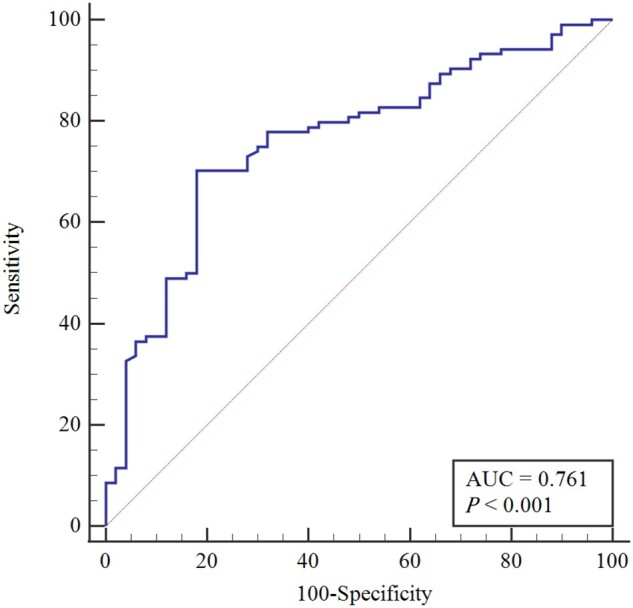
Predictive power of serum exosomal miR-144-3p for identifying active disease in patients with CD. Receiver operator characteristic curve analysis showed an AUC of 0.761 for endoscopic active disease in CD patients (cut-off value, 3.11, *P* < 0.001). AUC, area under the receiver-operating characteristic curve; CD, Crohn’s disease.

Circulating total miR-144-3p was not significantly correlated with CDAI (ρ = −0.037, *P* = 0.649) or SES-CD (ρ = 0.131, *P* = 0.091); however, serum exosomal miR-144-3p levels were positively correlated with CDAI (ρ = 0.343; *P* < 0.001) and SES-CD (ρ = 0.547, *P* < 0.001; Figure 2). Serum exosomal miR-144-3p levels were positively correlated with CRP (ρ = 0.332, *P* < 0.001) and ESR (ρ = 0.289, *P* < 0.001; Table 2). ROC analysis showed that circulating exosomal miR-144-3p had a high diagnostic accuracy for endoscopically active disease in CD patients (AUC, 0.761; 95% confidence interval [CI], 0.685–0.826; *P* < 0.001; Figure 3). The cut-off value for mucosal lesions was 3.11, with 70.2% sensitivity (95% CI, 60.4%–78.8%), 82.0% specificity (95% CI, 68.6%–91.4%), 89.0% positive predictive value (PPV) (95% CI, 82.1%–95.9%), and 56.9% NPV (95% CI, 45.2%–68.6%).

**Table 2.** Correlation analysis for various clinical indices

Index	miR-144-3p		CRP		ESR	
	ρ	*P*-value	ρ	*P*-value	ρ	*P*-value
CDAI	0.343	<0.001	0.440	<0.001	0.402	<0.001
SES-CD	0.547	<0.001	0.479	<0.001	0.409	<0.001
Rutgeerts score	0.478	<0.001	0.357	0.002	0.302	0.009
CRP	0.332	<0.001	–	–	–	–
ESR	0.289	<0.001	–	–	–	–

CD, Crohn’s disease; CDAI, CD-activity index; CRP, C-reactive protein; ESR, erythrocyte sedimentation rate; miR-144-3p, expression of serum exosomal miR-144-3p; SES-CD, simple endoscopic score for CD; ρ, Spearman rank correlation coefficient.

### Association of serum exosomal miR-144-3p levels and penetrating disease behavior

**Figure 4.**
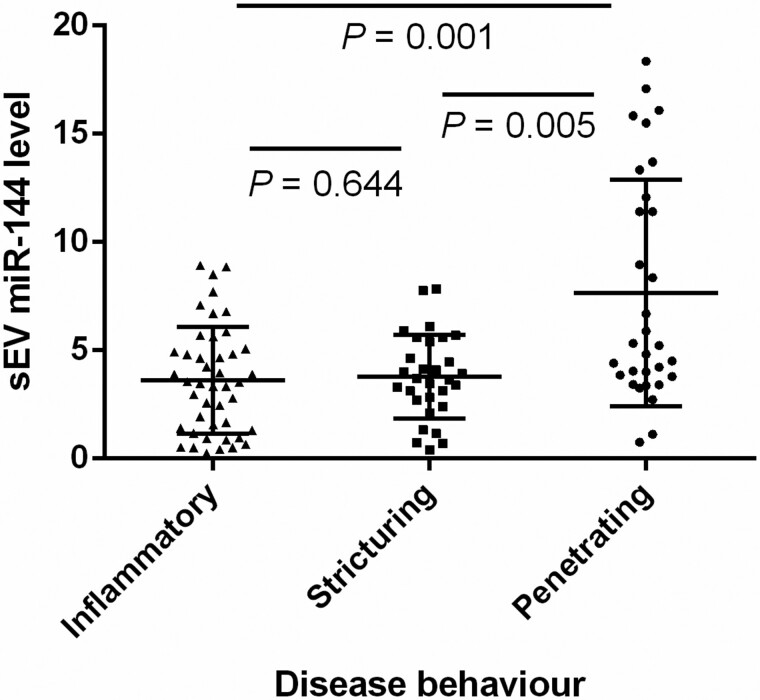
Expression of serum exosomal miR-144-3p in Crohn’s disease patients with endoscopic active disease with different disease behaviors. The level of serum exosomal miR-144-3p was significantly higher in patients with penetrating behavior than that in patients with inflammatory or stricturing behavior; sEV, small extracellular vesicle.

Due to the close relationship between serum exosomal miR-144-3p and disease activity, we evaluated the predictive value of the former for disease behavior and disease location in patients with mucosal active disease. The expression of serum exosomal miR-144-3p was significantly higher in patients showing penetrating disease behavior than in those showing inflammatory (*P* = 0.001) and stricturing disease behaviors (*P* = 0.005); no significant difference was observed between patients showing inflammatory and stricturing disease behaviors (Figure 4). Relative serum exosomal miR-144-3p levels of >8.92 were found to have 100% specificity (95% CI, 95.1%–100%) and 100% PPV (95% CI, 95.1%–100%) for the penetrating phenotype in patients with active CD with 33.3% sensitivity (95% CI, 17.3%–52.8%), 78.7% NPV (95% CI, 70.3%–87.2%), and an AUC of 0.719 (95% CI: 0.622–0.803). Serum exosomal miR-144-3p levels were not associated with the disease location (*P* = 0.078).

### Accuracy of serum exosomal miR-144-3p levels for identifying endoscopic recurrence

**Figure 5.**
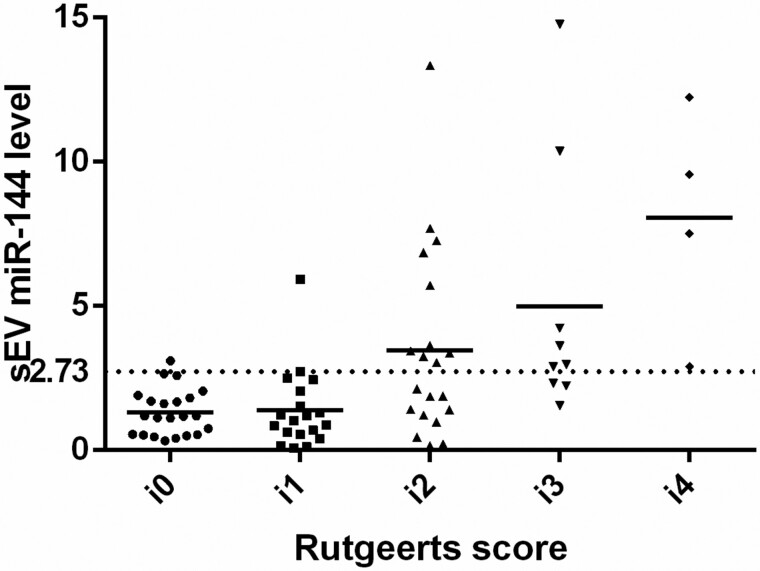
Expression of serum exosomal miR-144-3p in patients with Crohn’s disease after intestinal resection. The dotted line shows the cut-off value of 2.73. The majority of patients with expression of serum exosomal miR-144-3p more than the cut-off point of 2.73 were in endoscopic active stage (Rutgeerts score ≥ i2). sEV, small extracellular vesicle.

**Figure 6.**
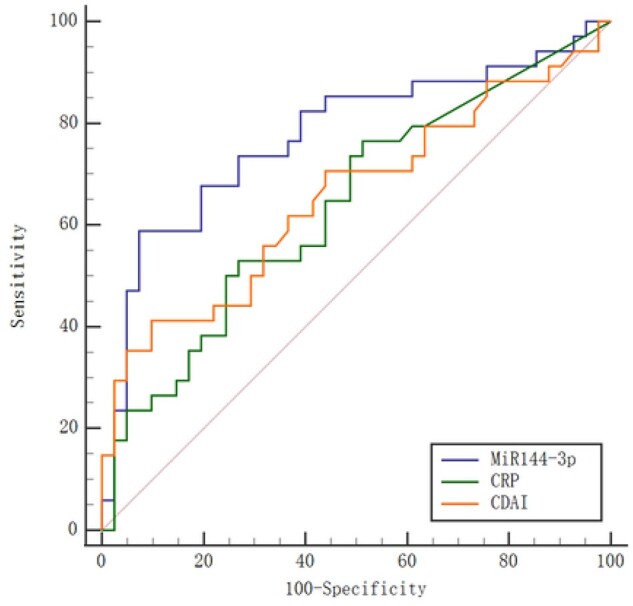
Receiver-operating characteristic curves of serum exosomal miR-144-3p and clinical indices identifying endoscopic recurrence. Performances of serum exosomal miR-144-3p in detecting endoscopic recurrence compared with CRP and CDAI. CRP, C-reactive protein; CDAI, Crohn’s disease-activity index.

Demographic characteristics of the 75 CD patients in Cohort 2 who underwent intestinal resection are shown in Table 3. At 18 months after surgery, all patients had undergone a colonoscopy and 45.3% experienced endoscopic recurrence. Serum exosomal miR-144-3p levels significantly decreased after intestinal resection (2.16-fold; *P* < 0.001); however, elevated expression of exosomal miR-144-3p was found in patients with endoscopic recurrence, with a fold change of 2.52 (endoscopic recurrence/remission; *P* < 0.001; Figure 5). Serum exosomal miR-144-3p levels were also positively correlated with the Rutgeerts score (ρ = 0.478; *P* < 0.001; Table 2). To identify patients with endoscopic recurrence, ROC analysis revealed that exosomal miR-144-3p had an AUC of 0.775 (95% CI, 0.664–0.864), which was significantly higher than that of the inflammatory marker CRP (0.639 [95% CI, 0.520–0.747]; *P* = 0.030) and ESR (0.591 [95% CI, 0.471–0.703]; *P* = 0.192; Figure 6). The optimal threshold was 2.73 at 58.8% sensitivity (95% CI, 40.7%–75.4%), 92.7% specificity (95% CI, 80.1%–98.5%), 87.0% PPV (95% CI, 72.1%–98.6%), and 73.1% NPV (95% CI, 60.6%–85.5%; Table 4).

**Table 3.** Demographic and clinical characteristics of 75 patients with CD in Cohort 2

Characteristic	Value
Male, *n* (%)	52 (69.3)
Age, years, median (IQR)	31.0 (25.0–43.0)
Disease duration, months, median (IQR)	48.0 (36.0–111.6)
Resection type, *n* (%)	
Ileocecal	32 (42.7)
Isolated ileal	19 (25.3)
Subtotal colectomy	18 (24.0)
Ileocecal and sigmoid	4 (5.3)
Ileocecal and small bowel	2 (2.7)
Disease phenotype, *n* (%)	
Inflammatory (B1)	5 (6.7)
Stricturing (B2)	25 (33.3)
Penetrating (B3)	45 (60.0)
Perianal disease, *n* (%)	29 (38.7)
Disease location, *n* (%)	
Terminal ileum (L1)	24 (32.0)
Colon (L2)	5 (6.7)
Ileocolon (L3)	46 (61.3)
Upper gastrointestinal disease (L4)	5 (6.7)
Endoscopic recurrence within 18 months, *n* (%)	34 (45.3)
Post-operative medication, *n* (%)	
5-ASA	4 (5.4)
Azathioprine	39 (52.0)
Mercaptopurine	1 (1.3)
Methotrexate	5 (6.7)
Thalidomide	13 (17.3)
Anti-TNF-α therapy	13 (17.3)

CD, Crohn’s disease; IQR, interquartile range; 5-ASA, 5-aminosalicylic acid; TNF, tumor necrosis factor.

**Table 4.** Receiver-operating characteristic analysis of endoscopic recurrence in 75 CD patients after intestinal surgery

Index	Cut-off	AUROC (95% CI)	Se (95% CI)	Spe (95% CI)	PPV (95% CI)	NPV (95% CI)	+LR	–LR	*P*-value
miR-144-3p	2.73	0.775 (0.664–0.864)	58.8 (40.7–75.4)	92.7 (80.1–98.5)	87.0 (72.1–98.6)	73.1 (60.6–85.5)	8.04	0.44	<0.001
CRP	2.57	0.639 (0.520–0.747)	52.9 (35.1–70.2)	73.2 (57.1–85.8)	62.1 (43.3–80.9)	65.3 (50.9–79.5)	1.97	0.64	0.030
ESR	27	0.591 (0.471–0.703)	41.2 (24.6–59.3)	85.4 (70.8–94.4)	56.0 (35.1–76.9)	60.0 (45.9–74.1)	2.82	0.69	0.192
CDAI*	150	–	44.1 (27.2–62.1)	78.1 (62.4–89.4)	62.5 (41.6–83.8)	56.1 (42.8–69.4)	2.01	0.72	0.018
CDAI	183	0.656 (0.537–0.762)	41.2 (24.6–59.3)	90.2 (76.9–97.3)	77.8 (56.5–99.1)	64.9 (52.1–77.7)	4.20	0.65	0.018

AUROC, area under the receiver-operating characteristic curve; CD, Crohn’s disease; CI, confidence interval; CDAI, CD-activity index; CRP, C-reactive protein; ESR, erythrocyte sedimentation rate; miR-144-3p, serum exosomal miR-144-3p level; –LR, negative likelihood ratio; NPV, negative predictive value; +LR, positive likelihood ratio; PPV, positive predictive value; Se, sensitivity; Spe, specificity.

Cut-offs were the optimal threshold value, except for the cut-off of CDAI*, which was based on the clinically determined value.

## Discussion

In this study, we determined the clinical significance of serum exosomal miR-144-3p as a non-invasive biomarker in two independent cohorts of CD patients. We confirmed that exosomal miR-144-3p levels were significantly higher in patients with CD, particularly in patients with penetrating disease. Furthermore, serum exosomal miR-144-3p was strongly correlated with endoscopic activity and showed better performance than CRP in identifying endoscopic recurrence after intestinal resection.

CD is characterized by alternating periods of remission and relapse. Most patients with CD require intestinal surgery over the course of the disease. Therefore, adequate monitoring of recurrent inflammation with timely personalized treatment is essential to reduce the severity of the disease and the need for further surgery [3]. Various biomarkers have been tested to evaluate disease activity to reduce repeated invasive examinations such as endoscopies. Prior research has indicated that circulating cell-free miRNAs are attractive biomarkers for various pathological conditions [13]. Serum total cell-free miRNAs include miRNAs derived from exosomes and miRNAs that bind to proteins; the latter are mainly released passively by apoptotic and necrotic cells. In contrast, exosomal miRNAs are specifically selected, encapsulated in vesicles, and actively secreted to mediate biological processes, and thus are more disease-specific for certain pathological conditions [11]. Moreover, exosomes containing miRNAs are remarkably stable and can thus be collected, transported, and stored conveniently, whereas other serum miRNAs are rapidly degraded by RNases. Notably, the ratio of exosomal serum miRNAs to total serum miRNAs varies substantially between individuals, and the total serum levels of a respective miRNA are not correlated with its levels in exosomes [28]. Therefore, exosomal miRNAs may be more reliable biomarkers.

Previous studies have shown that exosomal miRNAs derived from immune cells and colonic epithelial cells regulate immunity and intestinal barrier functions during IBD [15]. Recently, exosomal proteins annexin A1, PSMA7, and exosomal lncRNA NEAT1 have been reported to be correlated with disease severity; thus, they may serve as IBD diagnostic biomarkers [37–39]. However, few studies have evaluated the potential of exosomal miRNAs as non-invasive IBD biomarkers. In the current study, we selected miR-144-3p from the microarray data of serum and colon-tissue samples. We compared the expression of total serum miR-144-3p and serum exosomal miR-144-3p between CD patients and healthy controls. We found that serum levels of total miR-144-3p were not associated with exosomal miR-144-3p levels; both were considerably higher in CD patients than in healthy controls. However, while exosomal miR-144-3p in patients with active CD showed a fold change that was 2.12 higher than in patients with endoscopic remission (*P* < 0.001), no significant difference was observed in the total serum miR-144-3p levels between the active and quiescent diseases. Moreover, serum exosomal miR-144-3p was positively correlated with CDAI and SES-CD, whereas the serum total miR-144-3p did not correlate with disease severity. Thus, we concluded that serum exosomal miR-144-3p was more reliable for CD monitoring than total serum cell-free miR-144-3p.

Unlike disease location, which may remain unaltered during the disease course, the behavior of CD may change over time from an originally non-complicating to a penetrating or stricturing behavior [40, 41]. Therefore, the determination of the complicated phenotype helps to identify high-risk patients and facilitates the administration of early intensive therapy. We found that circulating exosomal miR-144-3p levels were significantly higher in patients with penetrating diseases than in those with inflammatory and stricturing diseases (Figure 4). ROC analysis showed that a cut-off value of 3.11 identified endoscopically active disease with 70.2% sensitivity and 82.0% specificity, while a cut-off value of 8.92 yields a high specificity of 100% and a sensitivity of 33.3% for the penetrating phenotype. Therefore, extremely elevated expression of exosomal miR-144-3p may improve the detection of penetrating diseases.

Severe complications of CD are indications for abdominal surgery. The present study showed that the expression of serum exosomal miR-144-3p significantly decreased after surgery; however, these levels were elevated in patients with endoscopic recurrence than in patients with endoscopic remission. Moreover, ROC analysis showed that exosomal miR-144-3p had a higher AUC, which can help to identify patients with endoscopic recurrence than the inflammatory marker CRP at 58.8% sensitivity and 92.7% specificity (AUC, 0.775 vs 0.639; *P* < 0.001). Furthermore, Wright *et al*. reported low performance of CRP and CDAI for detecting endoscopic recurrence, which was consistent with our findings [5].

The miR-144 family includes miR-144-3p and miR-144-5p, which are encoded in the 13q31.3 chromosome, and mediates erythroid homeostasis, tumorigenesis, and immune functions [42]. The miR-144 family target genes such as zonula occludens 1, anoctamin-1, and STAT family genes, as well as signaling pathways, such as the PI3K/AKT, NF-κB, Wnt, and JAK/STAT pathways [43–45]. Dissanayake *et al*. reported that serum miR-144-3p is elevated during atopic dermatitis and plays a pro-inflammatory role by promoting the expression of human b-defensin-1 and SERPINB4 through activation of the NF-κB pathway [46]. miR-144 is aberrantly regulated in several types of cancers where it acts as a tumor suppressor or oncogene, depending on the tissue type [42]. Gaedcke *et al*. found that dysregulation of mucosa miR-144 is rectal-cancer-specific rather than colon cancer [47]. A series of studies have found that miR-144 could be a non-invasive biomarker in several cancers, such as fecal miR-144 in colorectal cancer, serum miR-144 in gastric cancer, and serum exosomal miR-144 in hepatocellular carcinoma [48–51]. However, exosomal miR-144 as a non-invasive marker of CD was examined for the first time in the current study. Recently, Rojas-Feria *et al*. reported that miR-144-3p was significantly induced in inflamed colonic mucosa compared with non-inflamed mucosa in patients with active CD [30]. However, the mechanism underlying the regulation of miR-144 in CD remains unclear and requires further investigation.

This study has some limitations. The microarray analysis for exosomal miRNA in our study included three healthy individuals and three CD patients. The small sample size may limit the discovery of novel miRNAs. Two miRNAs, namely miR-144-3p and miR-451a, showed significant difference (Supplementary Figure 1). MiR-451a was not upregulated in the colon tissue of CD patients and was not correlated with disease activity when validated in a larger sample size; therefore, only miR-144-3p was investigated in this study, which also limits the potential of using a panel of exosomal miRNAs to increase the predictive value. Further studies are required to investigate serum exosomal miRNAs in CD patients in a large sample size and explore a panel of exosomal miRNAs for CD monitoring. Moreover, although the upregulation of serum exosomal miR-144 indicates mucosal active disease, the miR-144 levels may not reflect the severity of the disease accurately due to their relatively weak correlation with CDAI (ρ = 0.343) and SES-CD (ρ = 0.547). Additionally, we did not evaluate fecal calprotectin, which has been shown to detect endoscopically active disease and post-operative recurrence. Further studies are needed to investigate the association between serum exosomal miR-144 and fecal calprotectin.

In conclusion, serum exosomal miR-144-3p levels may be valuable for monitoring CD. Extremely elevated exosomal miR-144-3p levels indicate the presence of penetrating complications. This approach is superior to using the inflammatory marker CRP for detecting early recurrence in CD patients after intestinal resection. Serum exosome miRNA is stable and easy to detect without invasive procedures and therefore may be useful in clinical practice. Further studies are needed to validate these findings in independent prospective cohorts.

## Supplementary Data

Supplementary data is available at *Gastroenterology Report* online.

## Authors’ Contributions

S.Z. and M.C. conceived of and supervised the study. Z.Z. supervised the study and provided necessary guidance. S.Z., M.C., and Z.Z. contributed equally to this work. P.C. conducted the experiments, performed patient follow-ups, and wrote the manuscript. S.H. analysed the data and revised the manuscript. Q.Y., K.C., Y.W., G.Z., and X.Z. collected serum samples and compiled clinical information. All authors have read and approved the final version of the manuscript.

## Funding

This work was supported by the National Natural Science Foundation of China [grant numbers 81630018, 82070538, and 81870374], Guangdong Science and Technology Department [grant number 2017A030306021], and Guangzhou Science and Technology Department [grant number 202002030041].

## Supplementary Material

goab056_Supplementary_Data
