# Supplementary figures and images for: Serum exosomal microRNA-144-3p: a promising biomarker for monitoring Crohn's disease
Source: Gastroenterol Rep (Oxf). 2021 Dec 23;10:goab056. doi: 10.1093/gastro/goab056 (PMC8973006; doi:10.1093/gastro/goab056)

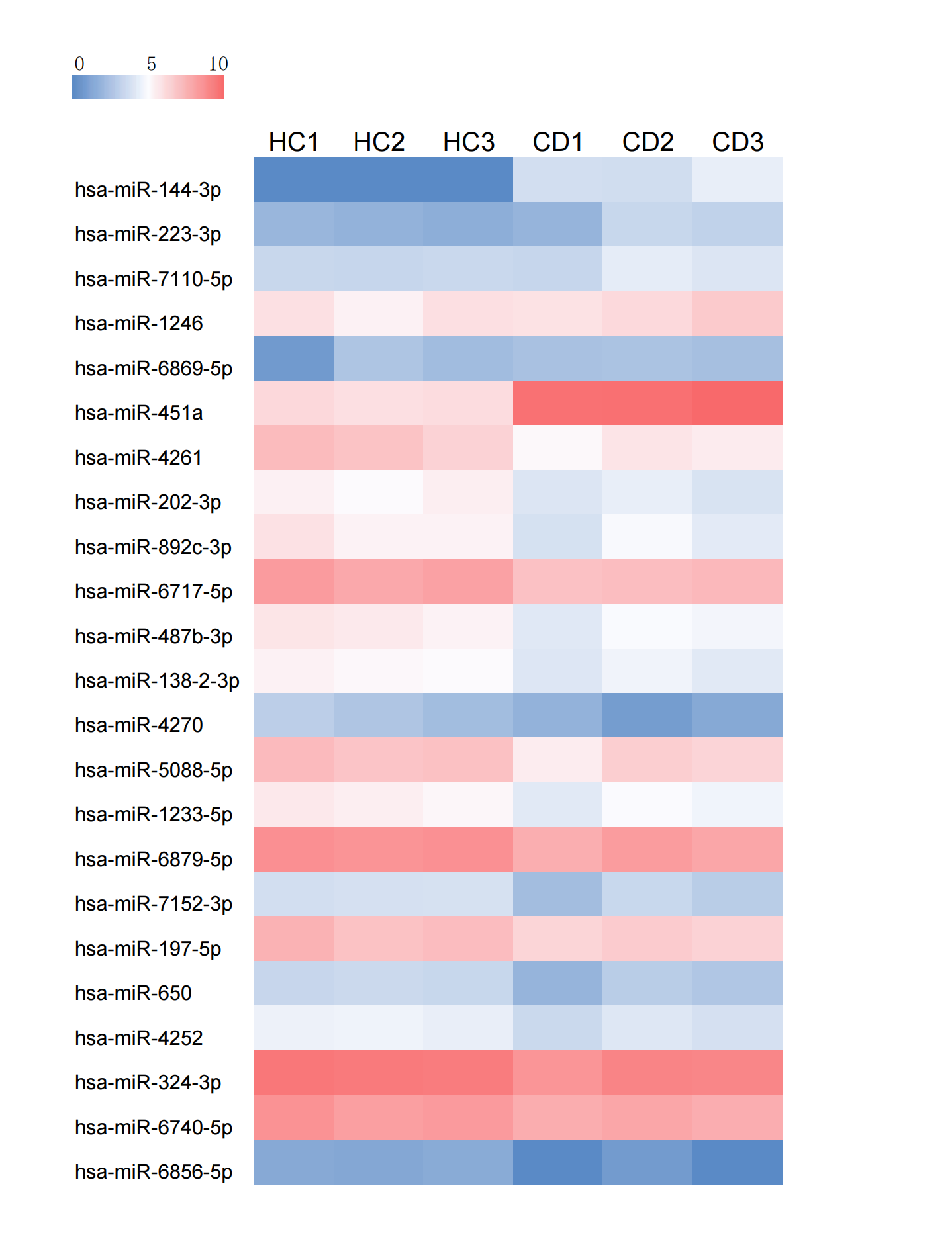

Supplement: goab056_Supplementary_Data [file goab056_supplementary_data.zip › Supplementary Fig 1.final version.tiff]

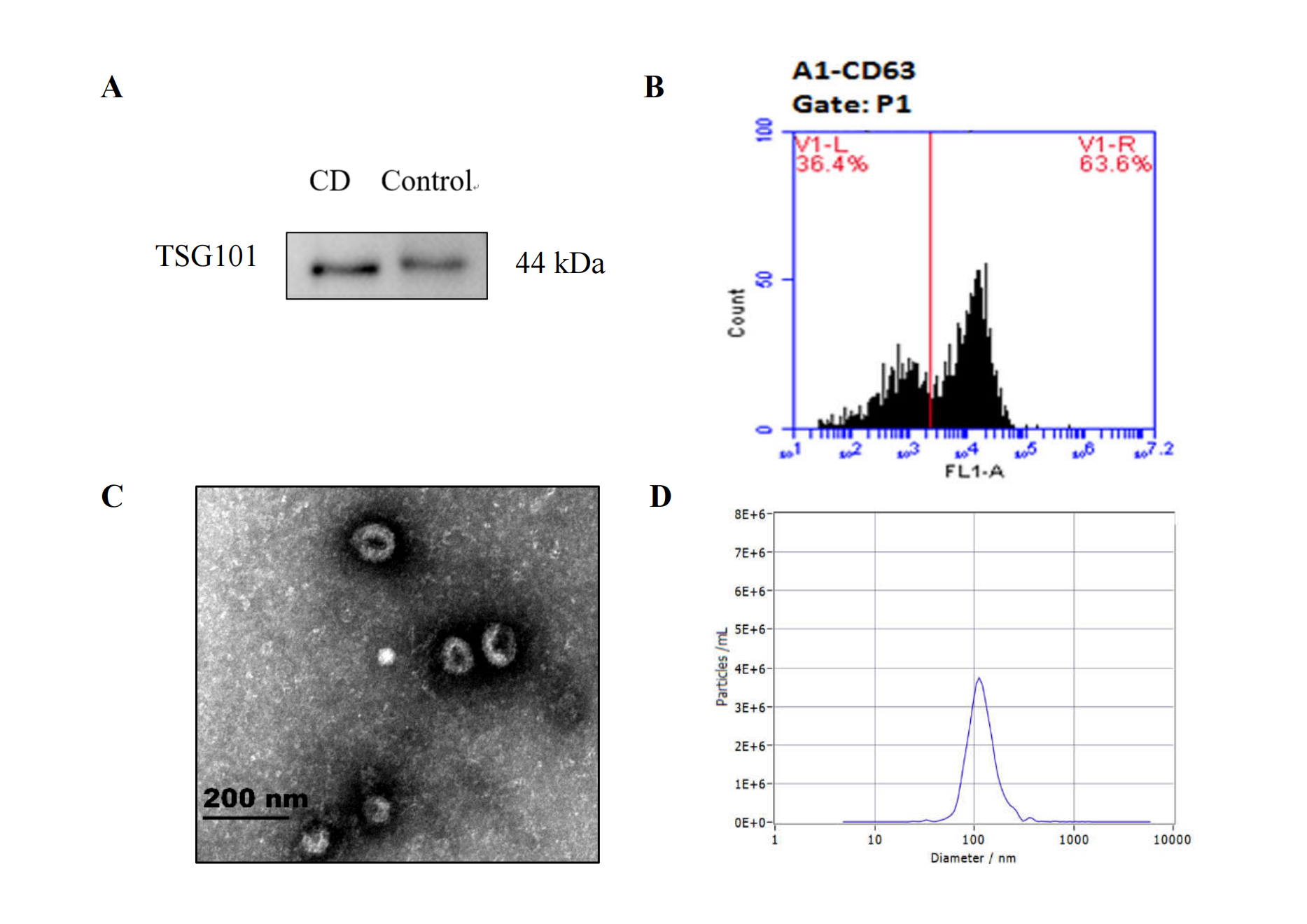

Supplement: goab056_Supplementary_Data [file goab056_supplementary_data.zip › Supplementary Fig 2.final version.tiff]
